# Sweet Potato as a Key Crop for Food Security under the Conditions of Global Climate Change: A Review

**DOI:** 10.3390/plants12132516

**Published:** 2023-06-30

**Authors:** Zagipa Sapakhova, Nurgul Raissova, Dias Daurov, Kuanysh Zhapar, Ainash Daurova, Andrey Zhigailov, Kabyl Zhambakin, Malika Shamekova

**Affiliations:** 1 Institute of Plant Biology and Biotechnology, Almaty 050040, Kazakhstan; zagipasapakhova@gmail.com (Z.S.); nraissova@gmail.com (N.R.); dias.daurov@gmail.com (D.D.); zhapar.zk@gmail.com (K.Z.); ainash.daurova@gmail.com (A.D.); zhambakin@gmail.com (K.Z.); 2 M. Aitkhozhin Institute of Molecular Biology and Biochemistry, Almaty 050012, Kazakhstan; andrzhig@gmail.com

**Keywords:** sweet potato, abiotic stress, drought, water stress, osmotic stress, mitigation of stress, drought tolerance, gene expression, transformation

## Abstract

Sweet potato is one of the most economically important crops for addressing global food security and climate change issues, especially under conditions of extensive agriculture, such as those found in developing countries. However, osmotic stress negatively impacts the agronomic and economic productivity of sweet potato cultivation by inducing several morphological, physiological, and biochemical changes. Plants employ many signaling pathways to respond to water stress by modifying their growth patterns, activating antioxidants, accumulating suitable solutes and chaperones, and making stress proteins. These physiological, metabolic, and genetic modifications can be employed as the best indicators for choosing drought-tolerant genotypes. The main objective of sweet potato breeding in many regions of the world, especially those affected by drought, is to obtain varieties that combine drought tolerance with high yields. In this regard, the study of the physiological and biochemical features of certain varieties is important for the implementation of drought resistance measures. Adapted genotypes can be selected and improved for particular growing conditions by using suitable tools and drought tolerance-related selection criteria. By regulating genetics in this way, the creation of drought-resistant varieties may become cost-effective for smallholder farmers. This review focuses on the drought tolerance mechanisms of sweet potato, the effects of drought stress on its productivity, its crop management strategies for drought mitigation, traditional and molecular sweet potato breeding methods for drought tolerance, and the use of biotechnological methods to increase the tolerance of sweet potato to drought.

## 1. Introduction

Rising global temperature levels as a result of climate change represent a significant challenge. Agricultural production is highly influenced by climatic factors, so it may be seriously affected in the near future if no actions are taken to accommodate and reduce the effects of abiotic stress on crops. Thus, there is an urgent need for the cultivation of crops that use water resources the most effectively. Sweet potato is considered to be one such crop, although its productivity is reduced under abiotic stress conditions. However, sweet potato has several advantages over other economically important crops that enable it to better address global food security and climate change issues, especially under the conditions of extensive agriculture seen in developing countries.

Sweet potato (*Ipomoea batatas*) is a basic foodstuff, fodder, and horticultural crop grown in tropical countries [1,2,3,4,5,6]. It ranks seventh in the world in terms of production [7,8,9]. It is a root vegetable crop of the *Convolvulaceae* family. *Ipomoea batatas* is the main staple crop. A few other *Convolvulaceae* species are localized, but many of them are noxious [10,11]. *I. batatas* is a herbaceous liana plant with alternating leaves and tubular flowers. Its edible tuberous roots may vary in shape and color depending on its variety and environmental conditions. These root tubers are usually long, and their skin color varies from white to purple [12,13]. *I. batatas* has a high WUE (water-use efficiency) and causes limited soil erosion during the rainy season, so it can be used as a cover crop as well as in the frost-free period of at least four months long [14]. Because of its high nutritional content and broad suitability for poor terrain, sweet potato is a prospective crop for preventing food shortages and enhancing food safety [1,3,6]. It also holds great promise for inclusion as part of a healthy diet in developing countries [1,6]. Less chemical pesticide and fertilizer are required for sweet potato cultivation in comparison to other crops [14].

*I. batatas* originates from the tropics of South America, where it has been cultivated for 5000 years [5,10,15]. Its global production is approximately 131 million tons year^−1^ on around 9 million hectares, having an average rated yield of 13.7 tons ha^−1^. Around 97% of the sweet potatoes grown worldwide are produced in developing countries. The crop is widely cultivated in Africa, Asia, and Latin America, with 52% of the crop being grown in China on an acreage of about 4.7 million hectares [16]. Today, thousands of sweet potato varieties are grown in all the tropical and subtropical climatic regions of the world [17,18]. Sweet potato is an important source of carbohydrates, vitamins A and C, fiber, iron, potassium, dietary fiber, and protein [11,19,20].

The growing recognition of sweet potato’s immense potential as a nutritional food for humans and animals has led to an increasing amount of research on its production and processing [11,21].

Sweet potato is a universal and hardly crop that grows best in warm, tropical climates with average temperatures of 24 °C. It is also an adaptable crop that produces large amounts of food per unit area and per unit time during short rainy periods, giving it an advantage over other staple foods [22,23]. Sweet potato has flexible planting and harvesting times, a short growing season, and a tolerance to high-temperature soils with low fertility [24], and it is not severely affected by pests or diseases [2]. In addition, growing sweet potato requires fewer labor resources compared to other crops, making it particularly suitable for small farms [17,24,25,26,27]. It can be used as a fast-rotating crop as a result of its wide ecological adaptation, drought resistance, and maturation period of three-to-five months [28,29].

In recent years, crops such as sweet potato have been introduced into regions with sharply continental climates, especially Kazakhstan, which is characterized by high abiotic stress risks (drought, salinity, high temperatures) as well as recurring low temperatures during the growing season [30].

## 2. Drought Tolerance Mechanisms of Sweet Potato

Salinity, low temperature, and drought are the three main environmental stressors that reduce the productivity of sweet potato worldwide [31]. Dryness limits sweet potato yields, resulting in an annual loss of 25% of the crop. The crop is especially susceptible to a lack of water during its establishment period, including at the vining stage and during root initiation. At the same time, one advantage of sweet potato is that it is drought-resistant after being rooted. This is why the yield potential (YP) of sweet potato is higher than that of other popular crops grown in developing countries.

Aside from its enhanced output, sweet potato’s excellent nutritional content is beneficial to farmers operating in drought-stressed areas.

At the same time, drought induces a number of morphological, physiological, and biochemical alterations in sweet potatoes, which can have a detrimental effect on their agronomic and economic output. For example, drought reduces root output, branching, leaf area index, stem height and length, stomatal closure, leaf size, and photosynthesis. Furthermore, it causes oxidative stress, which results in the creation of reactive oxygen species (ROS) that are harmful to plants. The activity and synthesis of other enzymatic and non-enzymatic antioxidant compounds, such as ascorbate peroxidase (APX) [32], glutathione reductase [33], catalase [34], superoxide dismutase (SOD) [33], carotenoids [35], ascorbic acid [36], glutathione, and tocopherols [34], increase under conditions of drought stress to either remove ROS or maintain them under tight control. Sweet potato’s drought resistance and tolerance are conditioned by the crop’s high level of antioxidants, which effectively controls the formation and effects of ROS [37,38].

A comprehensive study of the parameters connected with the drought response of sweet potato revealed several antioxidant enzymes that may manage the crop’s positive response during drought. In particular, assessing the activity of nitrate reductase (NR), free proline accumulation, and extracted chlorophyll concentration 60 days after planting was suggested as an effective method for screening genotypes associated with drought tolerance [33]. Another study found that orange-fleshed sweet potato varieties differed significantly in tuber weight, but their tuber quantity, beta-carotene content, starch content, and moisture content did not differ significantly. Further, the quantity, average weight, beta-carotene content, and starch and water contents of the tubers were not significantly affected by drought stress [35]. These orange-fleshed varieties have higher concentrations of Mg, Fe, Zn, Mn, Ca, and dietary fiber, whereas varieties with creamy flesh tend to have higher concentrations of starch and carbohydrates [33]. In addition, the high level of carbohydrates and important vitamins in sweet potato make them suitable for a healthy diet [14]. The secondary metabolites synthesized or activated under abiotic stressors are useful indicators for selecting the germplasm of sweet potato during breeding [39].

Agronomic evaluations of the creation of new plant varieties through traditional breeding in order to improve the genetic characteristics of the germplasm have focused generally on the yield of crops, with research on improving plant tolerance/resistance to abiotic/biotic stresses being less common. Considering the importance of the impact of abiotic stress on global agriculture, and to ensure global food security, new breeding programs have begun to be developed and implemented using knowledge of the physiological responses and molecular mechanisms of plants [40].

## 3. Physiological Responses of Plants to Drought and Water Stress

Drought affects several biochemical and physiological processes of plants, such as translocation, respiration, the uptake of ions, photosynthesis, nutrient and sugar metabolism, and phytohormones. Cell membranes can be destroyed, and leaf water potential can be diminished by drought. Furthermore, heavy drought causes the cessation of photosynthesis and metabolic disorders, and it can lead to the death of plants [40]. However, the drought sensitivity of plants depends on the degree and duration of the stress, the plant variety, and the development stage in which the drought occurs [41].

There are two known mechanisms that reduce the negative effects of drought stress in plants, namely stress avoidance and tolerance mechanisms. Stress avoidance refers to a plant’s ability to sustain the high water potential of its tissues under drought stress. Plants reach such levels by increasing their water uptake through deep root systems or by reducing their transpiration losses through thin or meaty leaves [40].

Drought is associated with changes in leaf anatomy and ultrastructure for most plant species [42]. Typical changes include leaf drying, reductions in stomata quantity, stomatal conductance, changes to cell walls, leaf hardening, leaf rolling, and the early induction of senescence. A study found that drought had a detrimental effect on sweet potato growth to the extent that no significant differences were observed among genotypes under severe drought conditions [43].

Different environmental conditions influence the growth, yield, and nutritional quality of sweet potato [39]. Through a water stress simulation experiment, a study found that water deficiency stress did not affect the tubers of sweet potato [39]. It has been emphasized that sweet potato is drought resistant and increases its level of secondary metabolites (for example, amino acids and β-carotene) under drought stress as a form of water stress protection [44]. These metabolites are useful to humans, as phytochemicals are conducive to a healthy lifestyle [44]. Although sweet potato is a drought-tolerant crop, it is drought-sensitive, especially at its early growth stages [39]. Delazari et al. [45] showed that sweet potato growth is severely stunted under drought conditions, which affects its yield. This corresponds to the conclusions of Martin and Jones [46].

Drought affects sweet potato structure not only at the tissue and cellular levels but also at the subcellular level. A study by Gouveia et al. assessed the physiological responses of sweet potato samples to conditions of water shortage. Sweet potato samples that had an improved WUE were found to be the most drought resistant [47].

In other similar studies, sweet potato samples showed the best physiological and biochemical responses to water stress treatment, showing in particular a higher ratio of above-ground to below-ground plant parts (root/shoot), lower total biomass loss, and lower stress index values [48]. In addition, the studied sweet potato samples showed a good phenotypic response, including water efficiency and nitrogen efficiency for growth and vital functions, as well as higher root mineral content, chlorophyll content index (CCI) values, and shoot nitrogen content [47]. Furthermore, all the samples reduced their biomass by 55.4%, thereby showing drought avoidance behavior under stress conditions. However, all the samples showed differences depending on their water distribution, chlorophyll level, and nutrient utilization. The sweet potato genotypes increased their WUE by +68.1% on average, and the highest water uptake occurred through transpiration. Furthermore, the samples’ chlorophyll content index values decreased by −5.3% as a result of a decrease in their photosynthetic rate. Their nitrogen efficiency ratios increased by +38.1%. Additionally, their nitrogen use efficiency increased by +54.4%. Their nitrogen harvest index values also increased, on average, by +2.9%. Overall, drought was shown to reduce the size of sweet potatoes (root/shoot ratio) as a result of investment in shoot development [47].

Another study found that plant signal transduction, phenylpropanoids, an isoquinoline alkaloids, and flavonoid biosynthesis play important roles in the regulation of the tolerance of plants to drought stress. According to the results of a transcriptomic analysis, the tolerance mechanisms of sweet potato varieties are very different, and occasionally some varieties respond oppositely. One drought-sensitive variety resisted drought stress by up-regulating signal production, whereas another drought-sensitive variety avoided drought stress by down-regulating isoquinoline alkaloid biosynthesis and nitrogen/carbohydrate metabolism. Moreover, on the one hand, some drought-tolerant varieties regulated flavonoid and carbohydrate metabolism or isoquinoline alkaloid biosynthesis and nitrogen/carbohydrate metabolism in response to stress; on the other hand, another drought-tolerant variety increased photosynthesis activity and carbon fixation processes. The high drought-tolerant variety was not affected by stress and responded to water deficiency by regulating the cell wall. These pathways are important indicators for selecting the breeding lines of sweet potato [41].

### 3.1. Chlorophyll Content Index

A study found that the chlorophyll content index values of sweet potato exposed to drought stress 60 days after planting did not decrease significantly compared to controls under drought conditions, although a decreasing trend was observed [33]. Zhang et al. [49] observed a decrease in CCI values over different periods (40 days, 60 days, 80 days, and 100 days) after planting under drought stress conditions. The Hernandez variety showed a slight increase in its CCI values in the control compared to in the high-stress conditions. Different sweet potato varieties expressed various CCI levels when exposed to the control treatment, including the high-stress treatment group 60 days after planting [33]. This can be explained by genetic differences among the varieties, as well as their photosynthetic activity.

Further, significant and strong decreases in CCI levels were detected 120 days after planting in all the genotypes under drought conditions. Similar data were obtained by Nikolaeva et al. [50], who observed a significant reduction in CCI levels when wheat plants were exposed to drought stress.

Another study found that some varieties of sweet potato (Monate, Resisto, and Bophelo) showed significant decreases in their CCI levels compared to the control. Drought affects the photosynthetic systems of plants and may cause the growth retardation observed in crown and stem development [33].

Chlorophyll breakdown can also affect the intensity of sweet potato’s antioxidant enzyme system, with relatively weak values having been recorded in previous studies. Heider et al. [51] considered the CCI to be a potential marker for selecting for heat tolerance.

### 3.2. Reactive Oxygen Species

Certain metabolites play leading roles in the adaptation of plants to a broad range of abiotic stressors [52]. The accumulation of osmolytes or compatible solutes, such as polyamines, free proline, trehalose, glycine betaine, and sugar alcohols, may protect plants against adverse environmental conditions.

A particular feature of sweet potatoes is that they contains sufficient quantities of β-carotene, vitamin C, and antioxidants [53]. These antioxidants provide the basis for the plant’s resistance to stressful conditions. Researchers have studied how the synthesis, activity, and levels of these secondary metabolites vary among sweet potato varieties [54,55,56,57].

In order to reduce the negative impact of abiotic stress, plants use different signaling pathways and react by changing their growth patterns, accumulating compatible solutes, activating antioxidants, and producing chaperones as well as stress proteins. ROS comprise radical and nonradical oxygen species generated by partial oxygen reduction [40]. A common occurrence for plants subjected to several abiotic stressors is ROS overproduction, which ultimately leads to oxidative stress [57]. This stress damages biostructures, such as proteins, lipid membranes, and nucleic acids, leading to plant cell death [40]. To reduce these damaging effects, plants have evolved enzymatic and non-enzymatic mechanisms that can minimize oxidative stress and help to increase their resistance to several abiotic stressors [44]. The activity of a plant’s antioxidant (enzymatic and non-enzymatic) system is an effective indicator of its drought tolerance [33].

### 3.3. Betaines

Betaines are non-protein amino acids that possess a quaternary ammonium group and a carboxylic group in their structure. These compounds effectively stabilize the quaternary structures of enzymes, complex proteins, and membrane systems, such as the photosystem 2 complex [58]. The synthesis of betaines is induced under different stress conditions, and their concentration is correlated with tolerance [59]. The accumulation of glycine betaine, the most widely studied betaine, results in the protection of plants against various abiotic stressors and increases their yields under non-stress conditions [60].

A study subjected embryogenic suspensions of sweet potato to an *Agrobacterium tumefaciens*-mediated transformation with a gene from spinach (*Spinacia oleracea*) called betaine aldehyde dehydrogenase (BADH). Transgenic sweet potato plants overexpressing this transgene were shown to have increased glycine betaine synthesis and an improved tolerance to multiple abiotic stress conditions, including oxidative, salt, and low-temperature conditions [61]. It has also been shown that transgenic sweet potato plants overexpressing the BADH gene from *Spinacia oleracea* chloroplasts have enhanced tolerance to osmotic, low temperature, and oxidative stressors [62].

### 3.4. Trehalose

Trehalose, a sugar consisting of two glucose molecules, functions as an osmoprotectant and plays a protective role against different adverse environmental conditions in both plants and animals [63]. It has also been implicated in the regulation of stomatal movement and water use efficiency in higher plants. Significant levels of trehalose in plant cells are vital for supporting growth under stressful conditions [64]. In plants, trehalose is synthesized in two stages by the enzymes trehalose-6-phosphate synthase (TPS) and trehalose-6-phosphate phosphatase (TPP). First, TPS synthesizes trehalose-6-phosphate, and then TPP catalyzes the dephosphorylation of trehalose-6-phosphate to trehalose [64]. A study isolated the TPS gene from *I. batatas* (*IbTPS*) and found that the overexpression of this gene in transgenic plants improved their resistance to salinity compared to control plants [65].

### 3.5. Polyamines

Polyamines are small polycations that play various important roles in all organisms. Polyamines, which are positively charged at physiological pH, interact with various negatively charged molecules, such as membrane phospholipids, nucleic acids, and certain proteins, which activate and stabilize them under abiotic stress [66]. Putrescine (a diamine), spermidine (a triamine), and spermine (a tetramine) are the most common polyamines in plant cells. They can be synthesized from positively charged amino acids, such as L-ornithine, L-lysine, and L-arginine [67]. A study found that transgenic sweet potato plants expressing the spermidine synthetase gene *FSPD1* from *Cucurbita ficifolia* showed higher levels of spermidine in their tissues and an increased tolerance to heat-mediated damage, chilling, and oxidative stress compared to wild-type plants [68].

### 3.6. Sugar Alcohols

Inositol is a well-known osmolyte, and its phosphorylated derivatives function as secondary messengers in signal transduction pathways under various stressors. A key limiting stage of myo-inositol biosynthesis is catalyzed by the enzyme l-myo-inositol-1-phosphate synthase (MIPS). A study isolated the *IbMIPS1* gene from *I. batatas* and found that its overexpression greatly impacted the salinity and water stress tolerance of transgenic sweet potato plants in field conditions [69].

### 3.7. Free Proline Content

Proline (Pro) accumulation is associated with abiotic stress tolerance mechanisms in plants. It helps to stabilize proteins and membranes as well as neutralize free radicals. Proline plays a role in supporting plants affected by stress conditions [38]. As an osmotic agent, proline helps to maintain the osmotic pressure between a plant’s extracellular and intracellular regions and protects plant cells from damage under osmotic stress conditions. The amount of free proline in plant cells increases significantly in response to various environmental stressors. Several studies have shown that the exogenous application of this amino acid may enhance plant resistance to drought [33,70]. The accumulation of free proline in transgenic plants can be achieved by enhancing its de novo biosynthesis [71] or by preventing the degradation of proline [72].

The key enzyme involved in proline biosynthesis is pyrroline-5-carboxylate reductase (P5CR). A study found that the overexpression of the *IbP5CR* gene isolated from *I. batatas* enhanced salt tolerance in transgenic sweet potato plants [73]. In a number of other studies, it has been shown that transgenic sweet potato plants with an enhanced tolerance to abiotic stress have a higher accumulation of free proline than wild-type plants exposed to the same stressors [52,74,75].

Laurie et al. [33] identified significant differences in free proline content among various genotypes. Proline accumulation increased from 2 µmol/g to 22 µmol/g under drought stress conditions compared to the control. An overall increase of up to five times that of the control group in plants exposed to the treatment conditions was observed 120 days after planting. This indicates that the plants were grown under drought conditions, which resulted in increased proline production either through free proline from the root system [76], an increase in enzyme production [77], or protein breakdown. The drought stress treatment conducted 60 days after planting found that the Bophelo sweet potato variety accumulated a higher level of proline compared to other varieties [33].

### 3.8. Ascorbate Peroxidase

Water stress has been found to cause a significant increase in APX activity, especially when treated with high drought stress. For instance, Zhang et al. [32] recorded an increase in APX activity and a defense reaction during the growth of sweet potato impacted by water deficiency stress. The APX activity increased by nine times under the drought treatment compared to the control. In addition, there was no difference in APX activity among sweet potato varieties. These findings are in accordance with the results of a study conducted by Dalton et al. [78], wherein a slight increase in APX activity was observed under drought stress conditions in wheat plants. Lu et al. [54] demonstrated that APX expression in sweet potato chloroplasts increases their drought tolerance and ability to recover from drought stress. Although most sweet potato varieties and breeding lines express moderate levels of APX activity under water stress, peroxidase enzymes are not the only antioxidant pathway used by sweet potato to reduce its ROS scavenger levels under water stress [33,49].

### 3.9. Superoxide Dismutase

A study observed an increase in the superoxide dismutase activity of different sweet potato varieties subjected to water stress. This ranged from 0.350 units/mg protein under the control conditions, to 0.85 units/mg protein under the drought treatment. These findings were similar to those made by Zhang et al. [49] and Masoumi et al. [79], who demonstrated increased SOD activity in soybean varieties under drought stress conditions. Differences among the varieties were observed at 60 and 120 days after planting in the severe-stress treatment group. The SOD activity values of the plants exposed to severe stress at 120 days after planting were also generally higher, which may have been related to the greater drought conditions experienced by the varieties, and also to an increase in diatomic oxygen recorded in the plant leaves [33].

### 3.10. Glutathione Reductase

A study found that glutathione reductase (GR) levels were increased in varieties subjected to drought stress at both 60 and 120 days after planting compared to the control group. Although the increase in GR was similar in the control and heavy stress treatment groups for each variety, the simultaneous increase in SOD activity confirmed the possibility of drought tolerance [80]. GR activity differed significantly among breeding clones because of drought stress. The GR activity ranged from 2 nmol NADPH min-1 mg protein-1 under the control conditions to 73 nmol NADPH min-1 mg protein-1 under the stress conditions. Among the varieties, significant differences in the activity of this enzyme were also found in the control treatment group at 60 days after planting. This may indicate the genotypic diversity of the varieties in terms of their GR activity under control conditions [33]. At 120 days after planting, the sweet potato variety Bophelo under drought conditions showed a significant increase in the activity of this enzyme. Furthermore, Masoumi et al. [79] observed a decrease in GR activity during drought experiments, indicating that tissue degradation may lead to decreased GR levels.

### 3.11. Nitrate Reductase

Xia et al. [81] investigated the activity of nitrate reductase (NR) under highly stressful drought conditions, finding that NR plays various roles in the regulation of NO_3_ assimilation and N-fixation, which are associated with the modulation of photosynthesis. The impact of drought was severe, meaning that no significant differences were observed among different genotypes. The rapid decrease in the enzyme’s activity in these trials was theorized to have a negative effect on plant growth. Reduced photosynthesis also has negative effects on nitrate reductase, which is consistent with the authors of [82]’s reasoning that stomatal conductance affects photosynthetic speed and therefore causes a decrease in the NR level of plants. A study found that the concentration of nitrate reductase ranged from 2.4 µmol NO_2_ g^−1^h^−1^ under control conditions to 0.0016 µmol NO_2_ g^−1^h^−1^ under drought conditions. No considerable differences in NR activity were found among varieties under conditions of severe stress at 60 or 120 days after planting when the activity was already at an extremely low level as a result of protein degradation [83]. Such a decrease in NR affects plant growth because nitrogen is an important precursor of the synthesis of secondary metabolites [33].

During breeding, the study of plant protection mechanisms under stress is important for the development of plant resistance and tolerance. This, in turn, is important for improving the productivity and yield quality of sweet potato and other crops [41]. Researchers have recommended field experiments using more sweet potato genotypes and in greater areas so that adaptation on a larger scale can be investigated [41,84]. Further, the determination of various physiological and biochemical parameters, such as sugar content and contents of secondary substances, such as phenols, which are related to water stress, is recommended for future sweet potato studies involving monitoring under drought conditions [40]. When selecting parents to cross for drought resistance, varieties with high antioxidant contents should be used to improve drought tolerance and avoid yield loss due to stress. Antioxidant levels should guide researchers in selecting and suggesting varieties for use in drought-prone regions in order to ensure sustainable agriculture and food availability.

## 4. Effect of Drought Stress on Yield

Water stress is a worldwide obstacle to high sweet potato yields because most sweet potato plants grow in semi-arid regions. Because of the complexity of the genetic and physiological mechanisms of water deficiency resistance, a greater emphasis on increasing our genomic understanding of sweet potato’s reaction to stress will help us develop strategies for maintaining its productivity under stressful conditions.

Laurie et al. [33] found that some genotypes of sweet potato had a high resistance to drought and a good yield index (%) under drought treatment. The same genotypes had higher geometric mean productivity (GMP), stress tolerance index (STI), and mean productivity (MP) values, indicating that they had high tolerance under both conditions. The study’s correlation analysis showed that YP and yield stress (YS) had highly positive correlations with the STI, MP, and GMP, and that they can be used as indicators for selecting drought-tolerant genotypes. Further, the stress tolerance index has been suggested as a useful indicator for areas under severe stress [85].

Agili et al. [28] identified several sweet potato genotypes with high productivity and good tuber quality, as well as tolerance to drought. The identified genotypes recorded high STI values and exceptionally low susceptibility index values. The study’s correlation analysis showed that YP and YS had very significant positive correlations with the STI, MP, and GMP. These parameters can be useful as indicators for selecting genotypes for tolerance.

In addition to its direct impact on yields, drought can also reduce the potential benefits of crop management practices, such as fertilizer application and pest and disease management. Drought requires additional periods of irrigation, which increases overhead production costs [86]. A lack of sufficient water for sweet potatoes, especially in their early stages of development, can lead to low tuber yields and a poor tuber quality. A prolonged period of drought can also significantly reduce sweet potato yield as well as the quality of its root tubers, causing great economic losses to farmers [47]. Therefore, it is necessary to improve the water use efficiency of agricultural crops, especially in areas with water shortages and where supplementary irrigation is required. In warmer crop cultivation areas, the effects of water stress are also increased by high temperatures [87].

Sweet potato yield is also significantly affected by stunted growth as a result of drought conditions. A study that exposed sweet potato to a medium level of water stress found that this had a negative effect on all of its characteristics, but that it still allowed for different genotypes to be distinguished; exposure to high stress had the opposite effect. The study showed [88] that a good yield is very much dependent on the plant maintaining a proper crown cover, stomatal conductivity, and stem length. Stem length and leaf area index measurements were strongly correlated with yield and can therefore be used as screening tools in future investigations.

The main objective of sweet potato breeding in many regions of the world, especially those affected by drought, is to obtain varieties that combine drought tolerance with a high yield. In this regard, the study of the physiological and biochemical features of certain varieties is important for the implementation of drought tolerance measures. In addition, it is necessary to carry out yield modeling to reduce the time and costs associated with repeating trials in larger areas.

## 5. Crop Management for Drought Mitigation

There are two options for crop management methods under conditions of water limitation: agronomic and genetic. The selection and improvement of genotypes adapted for a particular environment can be carried out with suitable equipment and through the use of selection indicators related to water deficiency tolerance. The creation of drought-tolerant varieties is a low-cost genetic management strategy that may be well-suited to small farms [89].

In order to develop an effective selection method for assessing water deficiency tolerance, indicators such as SOD activity, NR activity, and APX activity, stomatal conductance, leaf area, chlorophyll content, leaf water content, free proline content, and WUE should be considered at the beginning of the growing season. Various studies have assessed these parameters in a number of crops, such as sugar beet [90], potato [91], cotton [92,93], and wheat [94]. Such assessments save time and money when selecting a candidate genotype. These indicators should be used individually or in combination to develop suitable methods for the selection and breeding of genotypes.

Several approaches, such as the measurement of potential relative humidity, diffusion pressure shortage, chlorophyll stability index, and carbon isotope discrimination, have been used to assess the drought tolerance and WUE of crops [95,96,97,98,99]. However, these methods are time-consuming and, therefore, insufficient for screening large numbers of varieties. Earlier drought screening studies focused on drought as a whole without considering the separate component traits of drought tolerance [100,101]. These traits can be used as indicators for the selection of particular screening methods. They have also been used to develop screening method strategies, albeit with less success as a result of poor comprehension of the concept of drought tolerance and there being a lack of data on the genetic inheritance of stress tolerance in plants. In addition, plant protection mechanisms vary, making it difficult to use a single screening method to determine a plant’s stress tolerance. Nevertheless, some methods based on physiological and phenotypic methods, as described by Delazari et al. [45], have been used to determine genotype–environment interactions.

Although there have been few studies on the development of methods for evaluating the drought tolerance of sweet potato, a significant number of researchers have contributed greatly towards finding the optimal process for developing useful methods for selecting tolerant sweet potato germplasm. Ekanayake and Collins [87] used a sprinkler system from a line source to create drought conditions and investigated the effects of drought on the yield and leaf water potential of eight sweet potato varieties. Kubota [102], through conducting drought experiments using sweet potato pots, investigated the photosynthetic system, leaf surface development, stomatal conductance, leaf water potential, and soil water potential of the plants. Sung [103] found that sweet potato’s stomatal movement was not affected by water stress and that the plant’s nitrate reductase activity decreased as soil water potential reduced.

The optimal solution for developing a methodology to assess the drought tolerance of sweet potato is to grow the plant under field conditions where irrigation is applied without the interference of normal precipitation. Apart from field experiments, the adoption of strategies such as efficient water use, the proper selection of drought-tolerant genotypes, mass screening, conventional and molecular assisted selection (MAS), the exogenous application of hormones, the use of osmoprotectants on seeds or plants, and the development of genetic engineering methods for drought tolerance are recommended.

## 6. Breeding for Drought Tolerance

The most important factors limiting productivity must be considered when breeding crops. As shown above, drought is one factor that limits sweet potato yield, resulting in an annual yield loss. This is due to unfavorable changes at the morphological, biochemical, physiological, and molecular levels [89]. These changes are useful indicators for breeding and developing drought-tolerant sweet potato genotypes.

Sweet potato growing conditions differ greatly. Therefore, selecting the sweet potato germplasm based solely on the crop’s root yield for storage under optimal conditions may be disadvantageous for growers when soil conditions are poor. The impact of climate change around the world has made the choice of drought-tolerant varieties a priority for growers. Producers, especially small growers, need to consider several factors in order to achieve successful production [5]. Namely, they must choose varieties that can adapt to conditions such as poor fertilization, insufficient pest control, and, most importantly, infrequent irrigation water supply. Research plays an important role in selecting and developing the best varieties for the commercial market.

Traditional breeding has several limitations in terms of improving sweet potato properties. Most *I. batatas* varieties have reduced flowering and fertility or do not bloom. As a result of high levels of male sterility and self- and inter-specific incompatibility, sweet potato plants do not mix well in breeding programs [104]. The hexaploid nature (outcrossing polyploidy) of sweet potato also poses a challenge to its conventional breeding [10].

In addition, breeding sweet potato for drought tolerance requires an understanding of the consequences of drought stress, the availability of genetic diversity, and effective crossing and breeding methods that can lead to the identification and development of potential clonal varieties [105].

Drought is often a serious environmental barrier to the cultivation of sweet potato when it is grown in non-irrigated agricultural areas [106]. Different varieties may respond differently to limited quantities of groundwater. Thus, great importance must be given to breeding varieties with good characteristics under drought conditions.

The development of genetic management technology requires reliable, reproducible, simple, and rapid field and laboratory screening methods. These would enable researchers to identify drought-tolerance characteristics in the sweet potato germplasm and to include these in high-yielding varieties tolerant to drought stress [107].

Several researchers, such as Ekanayake and Collins [87] and Nedunchezhiyan [108], have conducted experiments assessing the effects of irrigation on the biological parameters of sweet potato [87] and the effects of mulching and pruning the top portion of the plant [108] to mitigate the negative effects of high temperatures. The results of such studies can be used to avoid water loss during sweet potato cultivation.

The use of drought and yield indices for the selection of derived germplasm for different production environments has produced encouraging results, and it should be carried out at the early stages of the breeding cycle [89].

Plant breeders must consider the crucial phases of plant growth and development. To increase sweet potato yield, particularly in Kenyan conditions, farmers have been advised to plant tubers early in the rainy season so that they are not subjected to water shortage conditions during the first four months of growth [109].

Omotobora et al. [110] researched the drought tolerance of 50 sweet potato genotypes under both laboratory and field conditions, finding 12 of them to be drought tolerant based on the number of days they took to wilt. Based on the above data, the authors recommended five of the most productive genotypes for breeding as parents in breeding programs for sweet potato drought tolerance.

Nhanala and Yencho [111] found that, at the phenotypic level, cultivated varieties were more tolerant to stress compared to wild species. In addition, they suggested that the storage of sweet potato roots may play a key role in the response of cultivated sweet potato to environmental stress. Because none of the wild type of *I. batatas* produce storage roots or have been found to be drought tolerant compared to cultivated sweet potato, it is likely that focusing on these species is the best option to improve the yield and drought tolerance of sweet potato.

Various mechanisms of plant tolerance to stress at the molecular and genetic levels appear to be interrelated, with environmental factors influencing the strength of their connections. Such molecular and genetic mechanisms have not been studied enough with regards to the phenotyping and genotyping of water stress resistance. In addition, epigenetic mobility and genetic element modifications are sources of variability in a plant’s stress resistance [112]. Despite the improvements that have already been made, there is still huge potential to further improve plant tolerance to abiotic stressors.

The above information may be useful for the general screening of sweet potato sensitivity or drought tolerance as well as for the adaptation of sweet potato to climate change using selection and breeding programs.

## 7. Molecular Breeding

Sweet potato is a hexaploid (2n = B1B1B2B2B2B2 = 6x = 90), and therefore, its gene expression has certain features. On the one hand, polyploidy introduces certain difficulties; on the other hand, the identification of genomes facilitates the study of gene expression in sweet potatoes.

As an alternative to traditional breeding, gene transfer technologies and genome-editing techniques are promising tools for the improvement of the environmental stress tolerance of sweet potato [113]. The reliable and stable transformation of sweet potato has been achieved using biolistic transformation, electroporation, and *Agrobacterium*-mediated transformation [52,114,115]. Several strategies have been implemented to improve sweet potato tolerance to abiotic stressors, including the transfer of genes encoding late-embryogenesis-abundant proteins, transcription factors (TFs), transport proteins, heat shock and cold shock proteins, enzymes leading to the accumulation of osmolytes and antioxidants, and hormone-related gene expression. A wide range of genes associated with resistance to abiotic stress have been characterized in *I. batatas*, raising the prospect of adopting cis-genic approaches [113,114,115,116]. Implementing genetic modifications to sweet potato crops in order to develop new varieties with improved tolerance to abiotic stress may reduce the negative effects of environmental stress as well as improve their water use efficiency and increase their productivity under unfavorable conditions, thus benefiting this economically important crop.

### 7.1. Induction of the Expression of Genes Encoding Stress Proteins

Plant heat shock proteins play key roles in conferring abiotic stress tolerance. Most of these stress proteins are molecular chaperones that assist in protein folding and transport and that prevent cellular functions from occurring under stressful conditions [117].

The overexpression of stress protein-encoding genes can promote transgenic plant survival under abiotic stress conditions. The overexpression of the cDNA gene At*P3B*, which encodes one of the ribosome-associated chaperones in *Arabidopsis thaliana*, improves the tolerance of transgenic plants [116]. Cell wall stabilization proteins have been shown to be important for mechanically stabilizing cells and membranes during osmotic stress conditions. The *Sap1* gene from *Xerophyta viscosa* encodes one such stress protein. A study exploited the *XvSap1* gene for the genetic engineering of sweet potato. The induced expression of this transgene resulted in transgenic plants that grew better than wild-type plants under drought stress conditions [75].

The genes of late-embryogenesis-abundant (LEA) stress proteins are expressed during seed maturation. LEA proteins are small and hydrophilic polypeptides that play essential roles in the abiotic stress response and stress tolerance of plants by acting as molecular chaperones [118]. The LEA14 protein, which is encoded by *IbLEA14*, induces enhanced tolerance to drought and salt stress, which is expressed in various tissues (leaves, stems, fibrous root, and embryogenic and non-embryogenic calli) under different water stress conditions. The expression of the *IbLEA14* gene is highly induced by water deficiency, salt stress, and ABA treatment in plants. The overexpression of *IbLEA14* in transgenic calli enhanced tolerance to drought stress. The *IbLEA14* gene regulates the increase in lignin, which has a crucial role in the drought stress response of plants. New studies will be required to study the role of lignin accumulation under stress conditions [119].

### 7.2. Induction of Expression of Genes Encoding Transport Proteins

The Na^+^/H^+^ antiporters are transmembrane proteins located in plasma or vacuolar membranes that operate to transport sodium out of the cytosol against a concentration gradient. These transport proteins maintain ion homeostasis, preventing the excessive accumulation of Na+ in the cytosol [120]. The overexpression of the *I. batatas* vacuolar Na^+^/H^+^ antiporter gene (*IbNHX2*) has been found to be associated with a significantly higher salt and drought tolerance in transgenic sweet potato plants than non-transgenic ones [121].

Similarly, the introduction of the Na^+^/H^+^ anti-transporter gene from *Arabidopsis thaliana* into the genome of sweet potato plants has been found to increase their resistance to cold and salt [62].

### 7.3. Preventing Oxidative Stress

The importance of ROS production as a response to abiotic stress has already been mentioned. However, it is also important to consider the role of ROS regulation at the molecular-genetic level. ROS are constantly generated in mitochondria and chloroplasts, as well as in response to different stress conditions. These compounds are highly reactive and dangerous, and they can directly damage cellular components and cause oxidative cell death [122]. A plant’s antioxidant defense system keeps it from experiencing excessive oxidative stress through ROS detoxification. The activation of ROS scavenging enzymes is a key response to water stress [61].

A study isolated the *IbNFU1* gene, which encodes the iron–sulfur cluster scaffold protein, from a salt-tolerant *I. batatas* variety and found that its overexpression resulted in a higher tolerance to salinity in transgenic sweet potato plants than in wild-type plants [74]. Iron–sulfur cluster scaffold proteins are ROS scavenging enzymes that play important roles in the assembly and transfer of Fe-S clusters, which are essential cofactors of proteins involved in energy metabolism [74].

A study used the genes of two ROS scavenging enzymes, Copper/Zinc (CuZn) superoxide dismutase (Cu/Zn-SOD) and APX, to increase the resistance of transgenic sweet potato plants to oxidative stress. The overexpression of these transgenes in *I. batatas* showed that the transgenic plants had a stronger ability to deactivate ROS compared to control plants [54].

### 7.4. Activation of Phytohormone Signaling Pathways

Abscisic acid (ABA) is a plant hormone that regulates a plant’s growth, development, and responses to various stressors. It plays a crucial role in enhancing plant resistance to abiotic stressors. The level of ABA in plants significantly increases under adverse conditions [36]. The molybdenum cofactor sulfurase enzyme catalyzes the formation of the sulfurylase form of molybdenum cofactor, which is required for the final step in the biosynthesis of ABA. The *LOS5/ABA3* gene, which encodes low osmotic stress 5 (LOS5) molybdenum cofactor sulfurase, is one of the key regulators of abiotic stress tolerance in plants [29]. The overexpression of the *Arabidopsis thaliana LOS5/ABA3* gene in sweet potato results in an enhanced salt tolerance [123].

A study showed that the α/β-hydrolase gene *IbMas,* which encodes the maspardin protein, regulates osmotic balance in *I. batatas*. Furthermore, the overexpression of this gene was shown to improve tolerance to salinity in cultivated sweet potato compared to wild-type plants [124].

### 7.5. Expression of Genes

The resistance mechanisms of plants are extremely complex, as plants must balance their growth and stress responses under stressful conditions. Because plants face many environmental stress factors, they have evolved complex resistance mechanisms for their balanced growth and development [49]. Several genetic and molecular studies have identified TFs, such as NAC, bZIP, WRKY, and AP2/ERF, to be the main regulators of plant resistance to abiotic stress [125,126,127,128]. In plant genomes, about 7% of coding sequences are assigned to TFs, and many of these genes are associated with an immediate–early response to abiotic stress [129].

Sugar will eventually be exported transporters (SWEET) are key transporters in sugar transportation. A total of 79 SWEETs have been identified in *I. batatas* (27 SWEETs), *I. triloba* (25 SWEETs), and *I. trifida* (27 SWEETs), and are named “*Ib*”, “*Itb*”, and “*Itf*”, respectively [130]. They are involved in the regulation of plant growth and development, the interaction of hormones, and biotic and abiotic responses to stress. In addition, several abiotic elements, such as the drought-responsive elements MYB [131], DREcore [132], and MYC [130], have been found in most *IbSWEETs*. Dai et al. [130] analyzed SWEET expression patterns using RNA sequencing data from *I. batatas*, *I. triloba,* and *I. trifida* under drought and salt treatment conditions, induced by polyethylene glycol (PEG) and NaCl treatments, respectively. Four *IbSWEETs* (2.1, 10.4, 15.1, and 15.7), six *ItbSWEETs* (2.2, 5.1, 10.2, 10.4, 15.1, and 15.3), and seven *ItfSWEETs* (2.1, 7.4, 10.3, 10.5, 15.1, 15.2, and 16.2) were found to be caused by both drought and salinity. Altogether, these results show that SWEET is expressed in different ways in response to various abiotic stressors in sweet potato and its two diploid relatives. Each SWEET gene plays a different, vital role in plant growth and development, hormone crosstalk, carotenoid accumulation, and responses to abiotic stress. This research provides valuable information on the structure and function of SWEET genes in sweet potato and its two diploid relatives. The results also indicate that high expressions of these *IbSWEETs* are involved in adaptations to abiotic stress in sweet potato. These *IbSWEETs* genes may be used as candidate genes to improve abiotic stress tolerance.

Lau et al. [133] demonstrated a gene expression profiling resource for drought stress experiments on sweet potato, showing its usefulness in creating functional genomics hypotheses. They identified a group of receptor-like kinases with leucine-rich repeats that are inhibited at 24 h post-stress but not at 48 h post-stress, suggesting that their reduced activity 24 h post-stress plays an important role in sweet potato’s response to dehydration. Interestingly, the downregulation of the *LHCSB6* and *SLAC1* single-copy orthologs was observed in both varieties at both time points [133,134]. These two genes encode effector proteins or the chlorophyll-binding component of photosystem II and the anion efflux protein of the closing cells, respectively, which have been shown to be very important for stomatal closure during drought stress in *Arabidopsis*. Thus, Lau et al. [133] presented them as strong candidates for hyperexpression experiments with sweet potato. Jointly regulated gene clusters involved in photosynthesis and the pentose phosphate pathway may contribute to making sweet potato’s chlorophyll content more resistant to drought [133,135]. On the other hand, a separate, co-regulated cluster of genes involved in the production of anthocyanin-containing molecules may help to increase the number of days before sweet potato permanently wilts.

Many studies on sweet potato have investigated changes in p-hydroxyphenylpyruvate dioxygenase (HPPD) gene expression levels induced by other abiotic stressors during oxidative stress [136,137]. Kim et al. [136] confirmed that the *IbHPPD* gene affects sweet potato’s resistance to various stressors. Transgenic sweet potato plants overexpressing *IbHPPD* were more resistance to herbicides, salinity, drought, and oxidative stress than non-transgenic plants [136,137].

Genes encoding TFs have attracted significant attention in recent years because of their ability to activate drought stress-resistance genes [138]. DREB/CBF class TFs have been shown to be effective in conferring drought tolerance [139,140,141]. Apart from the progress made in the study of the expression of stress-related transcription factor genes in transgenic plants, several reports have indicated that changes in hormonal homeostasis caused by the expression of isopentenyl transferase (IPT), a key enzyme in cytokinin biosynthesis and a promoter of the senescence-associated receptor protein kinase (PSARK), lead to an increased drought tolerance [142,143,144].

In recent years, the number of superfamily members with a domain-unknown function (DUF) has increased rapidly as research has progressed [145]. Although some genes in the DUF gene family have been identified, many *DUF668* members are still unknown [145]. The domain with unknown function 668 (*DUF668*) is a gene family that plays a vital role in plant responses to negative stressors associated with forcing. However, the function of the *DUF668* gene family in sweet potato has not been fully studied [145]. A study of transcriptome expression profiling showed that many genes from *DUF668* show specificity and differential expressions under cold, heat, drought, salt, and particular hormonal conditions in sweet potato (ABA, gibberellic acid, and indole-3-acetic acid). The study’s expression analysis showed that *IbDUF668-6, IbDUF668-7, IbDUF668-11*, and *IbDUF668-13* play important roles in the response of sweet potato to drought and salt stresses. The results suggest that the *DUF668* gene family is involved in drought and salt tolerance in sweet potato and also provide key information about the mechanism of *DUF668* genes in plants [145].

The WRKY TF family, initially isolated from sweet potato and named Sweet Potato Factor1 [146], forms one of the largest plant transcription factor families. The detection of the differential and remarkable expression profiles of *IbWRKY* under exposure to abiotic stressors and hormones is needed to clarify the signaling pathways associated with sweet potato responses to abiotic stress. Multiple *IbWRKYs* induced by stress may be closely related to the transcriptional regulation of abiotic stress responses in sweet potato, and a variety of interactions among *IbWRKYs* have been identified. The complex co-expression of *IbWRKYs* in response to abiotic stress is, therefore, predictable [147]. Further verification of the specific functions and regulatory mechanisms associated with the superior *IbWRKY* candidate genes involved in drought tolerance is also needed.

GRAS plant-specific TFs play key roles in a variety of unfavorable environmental conditions. Zhang et al. [32] studied 72 putative *IbGRAS* genes of sweet potato that had an irregular distribution and were isolated on 15 chromosomes and divided into 12 subfamilies. An RNA-seq analysis under salt stress conditions and the qRT-PCR determination of 12 selected *IbGRAS* genes demonstrated their significant and differing induction characteristics under exposure to multiple abiotic stressors (salinity, drought, heat, and cold) and hormonal influences (ABA, 1-aminocyclopropane-1-carboxylic acid, and salicylic acid). Consequently, the *IbGRAS* gene promoter regions were shown to contain several cis-acting elements related to stress and hormones. Among these, *IbGRAS71*, a potential candidate for plant tolerance selection, was characterized as having transactivating activity against yeast. The results of this study laid the foundation for the further functional identification of *IbGRAS* genes, and many members can serve as potential regulators for molecular selection for sweet potato tolerance [32].

Phytochrome-interacting factors (PIFs) are key regulators of plant responses to different abiotic stressors. The expression of *IbPIF3.1* has been found to be induced by abiotic stressors, such as drought, salinity, cold, and heat, as well as biotic stressors, such as *Fusarium* wilt tolerance [148].

A study found that the overexpression of the orange gene (*Or*), carrying the same mutation, resulted in high total carotenoid and beta-carotene contents and an increased resistance to abiotic stress in sweet potato crops [149]. Another study found that the sweet potato orange gene (*IbOr-R96H*), which carries a single-nucleotide polymorphism responsible for replacing arginine with histidine at the 96th amino acid position, significantly increased the carotenoid content and antioxidant activity in the storage roots as well as the abiotic stress resistance in the storage roots and leaves of transgenic sweet potato plants compared to cells overexpressing the wild-type *IbOr* gene (*IbOr-WT*) [150].

Kim et al. [150] established transgenic sweet potato plants overexpressing *IbOr-R96H* under the control of the 35S promoter of the cauliflower mosaic virus (CaMV) through an Agrobacterium-mediated transformation. The carotenoid content was higher in the stocking roots of the *IbOr-R96H* plants than in the stocking roots of the non-transgenic plants. In addition, the *IbOr-R96H* plants showed a greater resistance to heat stress than the non-transgenic plants and *IbOr-WT*, possibly as a result of their higher DPPH radical removal activity and ABA content [136]. These results show that focusing on *IbOr-R96H* represents a promising strategy for the development of new sweet potato varieties with increased carotenoid content and an improved tolerance to abiotic stress. Overall, the *IbOr-R96H* gene can help us cope with global food and nutritional security problems as well as create sustainable agricultural practices under climate change.

Nawiri et al. [151] used *Agrobacterium tumefaciens*-mediated transformation to regenerate transgenic sweet potato IPT lines. The overexpression of the IPT gene resulted in a significant increase in the drought tolerance of sweet potato, demonstrating the potential for developing drought-resistant varieties of this crop. The pathway involves the transfer of an isopentenyl group from dimethylallylldiphosphate to N6 adenosine monophosphate, resulting in the formation of isopentenyladenosine-5-monophosphate (iPMP). This reaction is catalyzed by dimethylallyl diphosphate and adenosine monophosphate isopentenyl transferases [152].

Tang et al. [137] identified 389 DEGs (differentially expressed genes) in response to drought stress. Using proteomic analysis, they further identified 1168 DEPs (differentially expressed proteins) under a drought treatment. These DEGs and DEPs were found to be associated with carbon, phenylalanine, starch, and cellulose metabolism, as well as heat shock proteins. Additionally, the study’s correlation analysis found 6607 co-expressed genes and proteins under the drought treatment.

**Figure 1:**
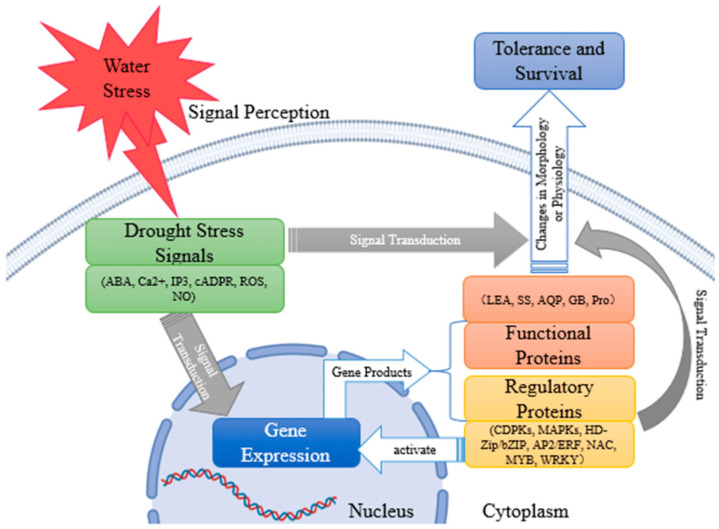
Plant tolerance mechanisms to drought stress conditions [156].

Overall, plants produce signal molecules, such as abscisic acid (ABA) [36], Ca2^+^ [38], inositol-1, 4, 5-triphosphate (IP3) [69], cyclic adenosine 5′-diphosphate ribose (cADPR) [152], and NO [83], in response to water stress signals under drought conditions, which either directly or indirectly cause changes in plant morphology and physiology. Indirectly, downstream genes are expressed as a result of drought stress signals. Proline (Pro) [71], glycine betaine (GB) [61], soluble sugar (SS) [52], late embryogenesis abundant (LEA) proteins [118,119], and aquaporin (AQP) [38] are examples of functional gene products that can participate in plant metabolism and consequently influence plant status. By controlling signal transduction pathways or acting as transcription factors to control the expression of genes, regulatory gene products, such as NAC [125], bZIP [126], WRKY [127], AP2/ERF [128], MYB [131], calcium-dependent protein kinases (CDPKs) [153], mitogen-activated protein kinases (MAPKs) [154], and HD-zip [155,156], can cause changes in plant morphology or physiology and further enable plants to survive in drought conditions (Figure 1).

Numerous plant responses to drought are controlled by several genes with different functions. Many regulatory processes are initiated when plant cells lose water to help regulate cellular metabolism and induce changes in gene expression. Whether these genes perform an adaptive role under drought conditions is completely unknown and, therefore, requires further investigation.

## 8. Response of the Sweet Potato to Transformation

Our understanding of the genetic control of plant mechanisms in order to overcome abiotic stressors has advanced in recent decades. The identification and cloning of candidate genes has enabled private and public researchers to create plants that can tolerate the adverse effects of these stressors without affecting their yields [137,149,150,151].

Various experiments by Nawiri et al. [151] involved the alteration and regeneration of sweet potato types. Somatic embryogenesis was found to still be genotype dependent in terms of how often and how differently the sweet potato plants regenerated. These results support the previous work of Anwar et al. [157], which achieved the successful production of sweet potato transgenic plants from different varieties using selected calli and by initiating somatic embryo formation on modified media. This indicates that existing regeneration and transformation protocols are highly dependent on a given genotype’s in vitro response. Because the leaf explants responded better to modification and regeneration than the other explant components, somatic embryogenesis was found to be the best technique to regenerate sweet potato. This study demonstrates the great sensitivity of sweet potato tissue to mannose and the need for a lengthy culture time in order to observe mannose’s effects, because its lethal effects cannot be seen in the early stages of growth. Additionally, an effective transgenic sweet potato PSARK-IPT plant was developed by Nawiri et al. [151]. Because of drought, these plants’ aging process was slowed, and they surpassed wild species in terms of their water content, chlorophyll content, tuber development, and growth [151].

Genetic engineering methods are one class of methods used to increase the resistance of plants to several negative environmental factors simultaneously, which, in turn, increases the rate of proline biosynthesis and decreases the rate of its biodegradation in plant cells [158]. Excluding carbohydrates, proline is the osmolyte with the highest prevalence and content in plant tissues, and a positive correlation has been found between its level in plant tissues and the plant’s resistance to adverse environmental factors, such as intensive ultraviolet radiation, increased soil salinity, low and high temperatures, and osmotic and oxidative stress [159].

Plants may synthesize proline in the cytosol via either the ornithine or glutamate pathways. However, the glutamate pathway of proline synthesis is mainly activated during plant adaptation to various stressors [160]. The genes of all the enzymes involved in proline biosynthesis have been successfully used by genetic engineers to produce plants that are resistant to cold, soil salinity, and osmotic stress [71,74,161]. However, proline can accumulate in plants under stress not only through the activation of its synthesis but also through the inactivation of proline degradation [162]. The main enzyme involved in proline biodegradation in plant cells is proline dehydrogenase. The silencing of this enzyme gene has been found to result in the significant accumulation of proline in *Arabidopsis* plants, which increased their resistance to frost and elevated salt content in the environment [72].

Among the many gene transfer technologies, the use of agrobacterial transformation continues to be the most effective strategy, and it could be a potential extension of classical breeding through which foreign genes can be introduced into plants via genetic transformation [163].

Despite being the seventh most common food crop in the world and having a yearly yield of 115 million metric tons [164], the improvement of the properties of sweet potato using genetic engineering methods has not been conducted as often as it has been for other important crops [165]. This is due to a lack of data on the full genome sequencing of sweet potato, which is a hexaploid, as well as the fact that, for a long time, it was impossible to adjust the processes of its transformation and regeneration [166]. However, in 2016, the complete sequencing of one of the haplogenomes of sweet potato was performed [166,167], which prompted the development of the functional genomics of this important crop. In addition, genetic engineers have recently managed to optimize the processes of sweet potato transformation and regeneration [168].

The development of the diversity of the sweet potato germplasm is important for ensuring food security worldwide because sweet potato is an important food in developing countries that are characterized by high populations and persistent malnutrition [169,170,171]. The findings from various recent sweet potato studies, including genotyping and phenotyping studies, provide guidance for the breeding of germplasm that is tolerant to abiotic stressors and also support the development of genetic resources and germplasm diversity [172,173,174,175].

## 9. Conclusions

Drought severely affects the survival and yields of crops and also increases the costs of crop production. Today, research on plant drought tolerance is becoming increasingly important, as it focuses on minimizing the impact of global climate change on agriculture. Sweet potato has attracted much interest from researchers interested in addressing the problems of drought tolerance in plants. Recent achievements in sweet potato genomics and metabolomics highlight the potential for using new, fundamental knowledge to carry out the practical breeding of the crop. To achieve the faster development of drought-tolerant sweet potato varieties, a combination of traditional and new techniques will be required. At the same time, it is necessary to improve the agronomic techniques used for specific soil and climatic conditions. Among the basic food crops, sweet potato has the greatest potential for adaptation to semi-arid and drought-prone areas. Developing drought-resistant varieties of sweet potato will increase farmers’ profits by reducing their irrigation and related production costs. Therefore, this crop can ensure food security for the increasing population of the developing world.

## Data Availability

No new data were created or analyzed in this study. Data sharing is not applicable to this article.
